# U-TAG: Electromagnetic Wireless Sensing System for Robotic Hand Pre-Grasping

**DOI:** 10.3390/s24165340

**Published:** 2024-08-18

**Authors:** Armin Gharibi, Filippo Costa, Simone Genovesi

**Affiliations:** Department of Information Engineering, University of Pisa, 56123 Pisa, Italy; armin.gharibi@phd.unipi.it (A.G.); filippo.costa@unipi.it (F.C.)

**Keywords:** robot grasping, pre-touch sensing, RF sensors, passive tags, printed tags

## Abstract

In order to perform complex manipulation and grasp tasks, robotic hands require sensors that can handle increasingly demanding functionality and degrees of freedom. This research paper proposes a radiofrequency sensor that uses a wireless connection between a probe and a tag. A compact and low-profile antenna is mounted on the hand and functions as a probe to read a printed passive resonator on the plastic object being targeted, operating within a pre-touch sensing range. The grasping strategy consists of four stages that involve planar alignment in up-to-down and left-to-right directions between the probe and tag, the search for an appropriate distance from the object, and rotational (angular) alignment. The real and imaginary components of the probe-input impedance are analyzed for different orientation strategies and positioning between the resonator on the object and the probe. These data are used to deduce the orientation of the hand relative to the target object and to determine the optimal position for grasping.

## 1. Introduction

Grasping tools, carrying items, and placing objects are basic capabilities and common operations for robots and robotic manipulators in various fields in which robotic hands or grippers are used such as assembly, repair, or human–robot interaction activities. In these fields, robotic hands play an important role in many manipulative tasks between robots and graspable objects [1]. Some key considerations for a grasping task include object characteristics (size, shape, weight, and texture), environmental conditions (lighting, temperature, humidity, presence of obstacles, and workspace layout), sensing and perception capabilities, and grasping strategy. Grasping tasks comprise the actions of gripping and moving an object from one place to another. All these operations need high accuracy, high reliability, high speed, and high flexibility. However, they are conducted in an environment that the robot needs to be aware of to perform its task [2,3], and the sensing system plays a vital role in this regard. Many solutions have been developed to increase the functionality of robotic hands due to depth cameras becoming widely accessible and high-precision tactile sensors becoming less expensive [4,5,6]. However, sensor tasks and operative scenarios are becoming more and more varied while, even with multimodal sensor fusion, an accurate and reliable estimation of grasping positions for complex objects remains a challenge. These challenges include grasping transparent objects, non-canonical shapes, objects in dark or heavily cluttered environments, and unknown objects [7,8,9]. The grasping process involves several key steps: perception, object detection, grasp planning, reach planning, and grasping execution. During this operation, the robot first perceives the environment and identifies objects within it. It then decides which objects are suitable for grasping, selects optimal grasp points on their surfaces, plans its reach to the desired position and orientation for grasping, and, finally, closes its gripper or hand to securely grasp the object. Various sensing systems may be activated for each step of the process. Vision sensors are commonly employed as proximity sensors when the hand is not in close proximity to the object. The main reason for this can be the blind spot of these kinds of sensors at very close distances [10]. The main application of capacitive sensors is normally in the tactile range, although they have some limitations in sensing range and sensing of nonmetallic and non-grounded objects [11,12]. They are a novel sensing system based on acoustic aura, capable of comprehensive pre-touch and contact sensing across the entire surface. This system incorporates a single piezoelectric transducer integrated into a 3D-printed finger design and adjusts the robot gripper’s pose to align a target object at the center of both fingers. In [3], a vision-based grasping platform equipped with a parallel gripper and RGB-D camera achieves automatic robot grasp. This grasping system estimates the grasp, represented as the rotated bounding box only from the single visual-sensing modality (i.e., RGB or depth image). In [13], a capacitive sensor for the combined functions of proximity control and contact grasping-force detection is presented. A ring-shaped electrode can ensure that the electric field lines located at the edges, which are feasibly disturbed by the approaching object, lead to a ~100 mm proximity detection of the sensor. In addition, the gradual deformation of the pyramid microstructure between the top and bottom electrodes renders a sectionalized linear response to the applied force in the range of 0−12 N. Boroushaki et al. [14] present a robotic system that can retrieve RFID-tagged items also in non-line-of-sight or in fully occluded settings by using a camera, an antenna, and tagged objects. Smith et al. [15] present a series of experiments encompassing human-to-robot and robot-to-human handoffs, integrating electric field pre-touch to detect the “co-manipulation state” when both humans and robots are in contact with the same object. While these existing solutions have helped tackle the grasping challenge, there are still opportunities to enhance robots’ capabilities with additional sensor systems. Factors such as sensor performance depending on the shape and size of the target object, versatility in operating across different environments, the capacity to work in close proximity to objects, and the ability to orient with non-metallic objects, alongside considerations of simplicity, compactness, and cost-effectiveness, could be key areas for further improvement.

Radio Frequency (RF) sensors are adaptable devices that use radio waves to detect motion, distance, temperature, and objects. They work in diverse conditions like darkness and harsh conditions, providing real-time data without physical contact. These kinds of sensors have a wide range of applications, including radar for object detection, Radio Frequency Identification (RFID) for inventory tracking, and wireless networks [16,17].

This study presents a new wireless radio-frequency-sensing system that operates within a pre-touch sensing range for grasping plastic objects. Its objective is to orient the robot hand with the object in various directions by utilizing probe-resonator interactions. Consequently, irrespective of the object’s color, size, shape, or surface properties, the grasping operation becomes feasible by positioning and orienting the resonator appropriately on the object. This sensing system excels in providing close-range sensing capabilities, overcoming issues that optical sensors may encounter such as blind spots, reflections, or low-light conditions. Unlike capacitive sensors, the sensing system does not face limitations in detecting plastic or non-grounded objects. Another advantage is its multifunctionality, as it can detect linear motion, distance, and the angle between the hand and the object. The cost efficiency and ease of production are notable benefits of this sensing system. It is designed to be economically viable, making it affordable to produce and deploy. Preliminary investigations have been conducted for a sensing system for robotic grasping and localization in [18,19,20,21], whereas angular position monitoring based on passive resonators has been exploited in a far-field reading scenario in [22]. In [23], two different kinds of CWP antennas were introduced as probes to find the best design for the angular alignment of the hand with the object presented. In this work, the approach has been further developed to detect the object even in near-field scenarios. The robotic hand is then aligned in the desired location and distance from the object for grasping.

## 2. Addresses Problem and Design

**Figure 1:**
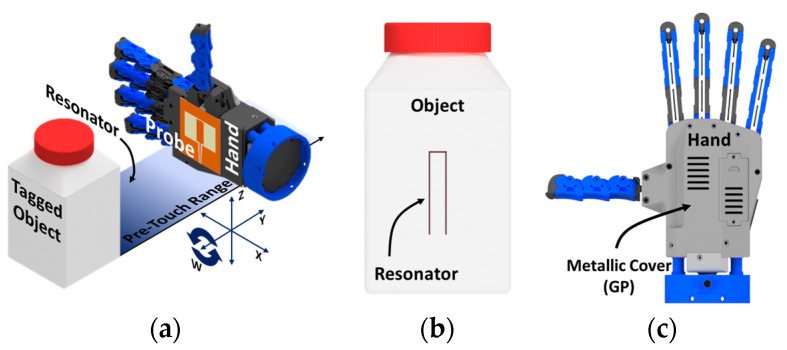
A 3D illustration of a robotic hand and an object in a grasping scenario, with (**a**) focusing on the internal components, (**b**) a front view of the object with a resonator, and (**c**) a back view of the hand with the ground plane.

The addressed scenario is illustrated in Figure 1, which comprises a robotic hand featuring an integrated probe, an object equipped with a specially designed resonator, and their alignment and grasping configuration across four different moving directions, (i.e., *X*, *Y*, *Z*, and *W*), where *W* represents the angular rotation (counterclockwise sense) around the *Y* axis, seen from the negative-toward-positive direction. Different operation frequencies have been chosen to provide a flexible range of work. The angular and planar orientation of the hand and the object is retrieved by observing the change in the impedance of the antenna probe, which will be modulated by rotating the hand around the axis perpendicular to the object and resonator and moving the hand on the parallel plane with the surface hosting the resonator printed on it [24,25]. The impedance behavior is also investigated by changing the distance between the hand and the object in all planar and angular positions. The proposed approach can provide the desired alignment between the probe and the resonator, as well as an estimate of the relative distance from the object to ease a successful grasp.

To achieve successful grasping of the object, it is essential to accurately determine its angular alignment with the hand. This necessitates the design of the appropriate probe and resonator for the task. Initially, a series of simulation studies were conducted using CST Microwave Studio for this purpose. The hand has been represented by a PTFE substrate (ε_r_ = 2.1) with a thickness of 45 mm. The hand’s backside is grounded to replicate the metallic back of the robotic hand depicted in Figure 1c. The object is a rectangular PTFE medicine box measuring 50 mm × 50 mm or 80 mm × 80 mm with a thickness of 1.6 mm. The substrate used for the antennas was FR-4 with (ε_r_ = 3.2).

In the setup of the simulation, an 8 mm distance was maintained between the hand and the object. The simulation subsequently progressed in 18-degree increments of angular rotation, spanning from −90 degrees to 90 degrees across the frequency range from 500 MHz to 5 GHz. The simulation scenario involved the alignment of the hand and object in planar directions, such that the resonator’s center point was positioned ahead of the probe’s center point. Additionally, the hand and object were oriented parallel to each other. To visualize the variation in probe-input impedance as the angle changes, the data points corresponding to each peak frequency of each specific probe are indexed. Subsequently, the impedance values at these selected frequencies are plotted against the corresponding angles. This method allows for a better understanding of how the impedance of the probe changes as a function of angle.

### Optimal Probe Resonator Configuration

**Figure 2:**
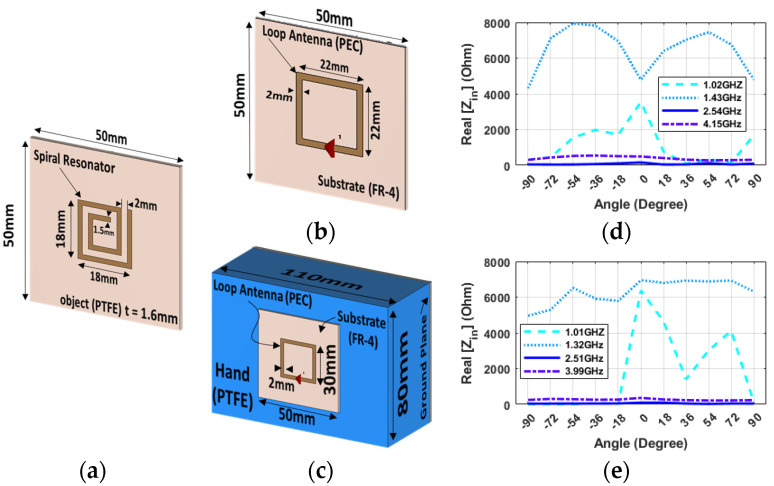
Spiral resonator configuration: 3D illustration for (**a**) the spiral resonator, (**b**) the loop antenna without hand, (**c**) the loop antenna on hand and near GP. The real part of Impedance (CST) at 8 mm versus different angles and frequencies for the spiral resonator in coupling with (**d**) the loop antenna without hand, (**e**) the loop antenna on hand and near GP.

In order to select the optimal topology for the probe and resonator, three different solutions are investigated. The initial candidate for the probe during the simulations was chosen based on a distance sensor that prominently features a spiral resonator (SR) label printed on a dielectric substrate (Figure 2a) and remotely interrogated via a small-loop antenna (Figure 2b) as a reader presented in [26]. By observing the change in the real part of the input impedance in four different peak frequencies of the loop and spiral resonator (Figure 2d), it can be observed that there is a potential ambiguity in estimating rotation angles of 0 or ±90 degrees since their values are close due to the intrinsic symmetry of the probe. For example, even though at the frequency of 1.43 GHz there is significant variation in the real part of impedance across different angles, the values are very close together at angles of 0 or ±90 degrees. Additionally, when the probe is integrated into the robotic hand (Figure 2c), its behavior becomes unstable, rendering the impedance ineffective for angular alignment goals (Figure 2e).

**Figure 3:**
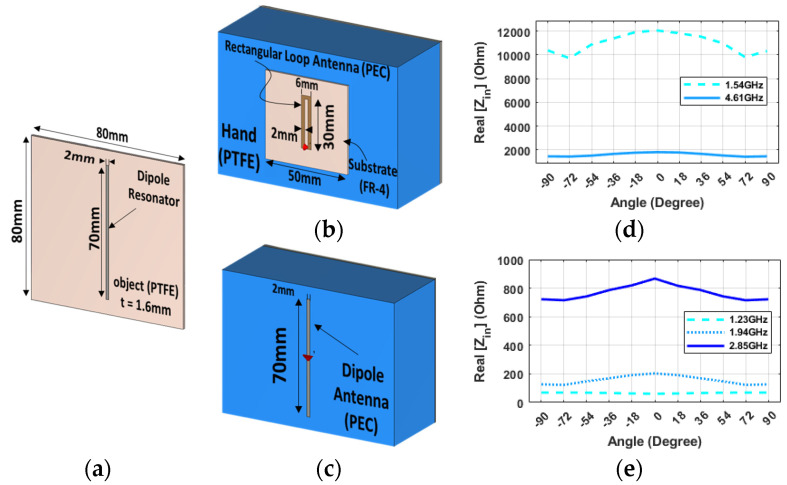
Dipole configuration: 3D illustration for (**a**) the dipole resonator, (**b**) the narrow-loop antenna on hand and near GP, and (**c**) the dipole antenna on hand and near GP. The real part of Impedance (CST) at 8 mm versus different angles and frequencies for the dipole resonator in coupling with (**d**) the narrow-loop antenna and (**e**) the dipole antenna.

To break the probe symmetry, a narrow rectangular loop antenna is employed together with a dipole resonator to maximize the difference between 90 degrees and zero degrees by maximizing the change of coupling between the resonator and the probe (Figure 3a,b). The narrow loop, in coupling with the dipole resonator, has two peak points at 1.54 GHz and 4.61 GHz. Its behavior could be suitable for angular alignment in both cases. However, the fluctuation in values between different angular alignments is low, and the curve is very smooth (Figure 3d).

**Figure 4:**
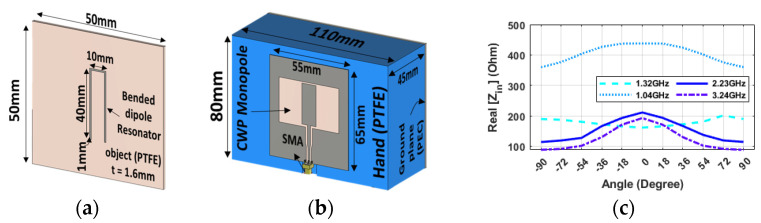
Hairpin resonator configuration: 3D illustration for (**a**) the U-shaped bent resonator, (**b**) CPW monopole antenna on hand and near GP, and (**c**) the real part of Impedance (CST) at 8 mm vs. different angles and frequencies for the U-shaped resonator in coupling with CPW monopole.

A better solution seems to be represented by a dipole probe (Figure 3d) on the robot hand. The system in Figure 3c comprises a half-wave dipole antenna on the hand palm and a plastic object with a metal strip. In coupling the dipole probe with a dipole resonator, three peak points were observed at 1.23 GHz, 1.94 GHz, and 2.85 GHz, respectively. As evident from Figure 3e, the 2.85 GHz frequency can be useful for the angular alignment of the hand with the object, as the impedance value is minimal when the hand is aligned at ±90 degrees. In contrast, at 0 degrees, the impedance value is maximum. Moreover, the curve is sharp enough to provide precise detection of the alignment angle, and all of the neighbor points near 0 degrees have a lower impedance value. As a further improvement, a hairpin-shaped tag that provides a much more compact form factor has been adopted to shrink the size of the resonator, thus requiring a smaller footprint of the tagged object (Figure 4a). And a more practical probe design based on a monopole antenna, which can be fed by an unbalanced line such as a coaxial cable, was developed in view of easing the implementation of the probe into the robotic hand, (Figure 4b). This configuration has four distinct peak points and offers two different frequencies for our goal, as well as sharper changes and a greater variation with changes in the angle (Figure 4c).

## 3. Experimental Verification

The working principle of this sensing system involves examining the change in coupling between a handheld probe and a fully passive chip-less resonator attached to a plastic object. Initially, the optimal design obtained from simulation results is fabricated, and the performance of both the probe and the tag is tested. To ensure a robust sensing output, the system focuses on changes in the real and imaginary parts of the input impedance of the probe and tag. The experimental analysis is conducted across various scenarios, with data classified into four sections: left-to-right alignment, up-to-down distance, and angular alignment. Due to the wide range of selected frequencies, achieving the desired alignment in each direction can be accomplished through multiple methods.

### 3.1. Fabrication

**Figure 5:**
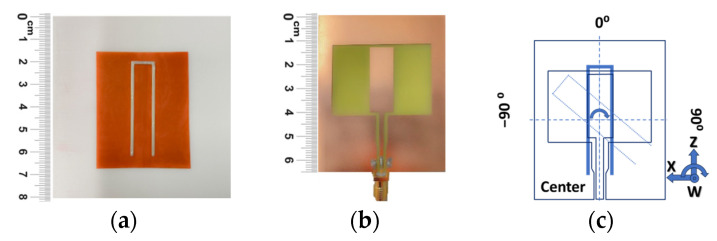
System execution (**a**) selected resonator attached to the target object, (**b**) CPW monopole probe, (**c**) overall diagram illustrating the positioning of the probe and resonator at the center in all directions (complete alignment).

Based on the analysis performed in the previous section, the employed resonator is a 1 mm-width bent monopole with a hairpin shape, printed on a flexible Kapton substrate (ε_r_ = 3.3) with a thickness of 0.15 mm. The overall length is equal to 90 mm, where both arms extend for 40 mm and are separated by 10 mm. This design ensures practicality and suitability for the intended application while maintaining a compact size to ease the application of it on the object. For the printing process, a VOLTERA V-One Flex 2 silver ink with high electrical conductivity and flexibility, suitable for the VOLTERA V-One circuit 3D printer, was employed to create the resonator (Figure 5a). The printed tag is placed on top of a fully filled PLA object with dimensions of 80 mm × 80 mm and a thickness of 2 mm, which will be considered the tagged plastic object for our purposes. The probe is a co-planar waveguide (CPW) monopole antenna with a total dimension of 55 mm × 65 mm, which is realized with a standard PCB process on an FR4 substrate (ε_r_ = 4.4, tg δ = 0.022) with a thickness of 0.15 mm. An SMA (subminiature Version A) connector is used to feed the probe (Figure 5b). The envisioned robotic hand measures 100 mm × 70 mm and has a thickness of 45 mm between the palm and the metallic rear cover. These components are then placed on the test platform to conduct experimental tests that help the sensing system align the hand and object in all directions (Figure 5c) for grasping the object.

**Figure 6:**
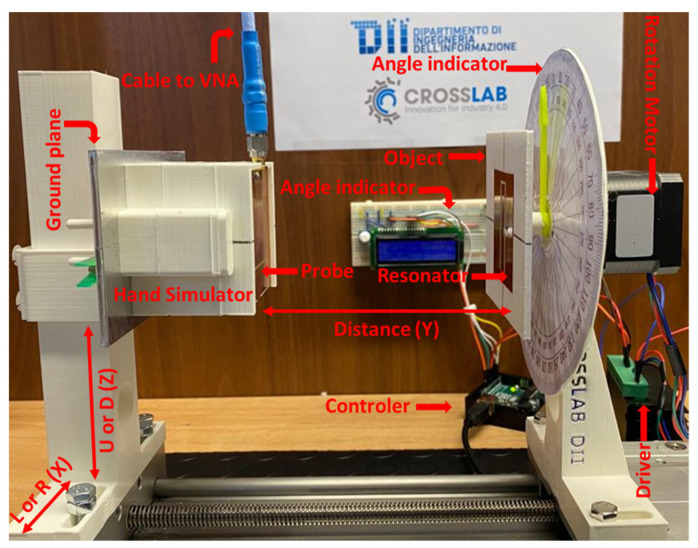
Experimental arrangement closely mirroring realistic conditions for evaluating and assessing system performance.

Figure 6 shows the experimental setup, including the moving axis and the placement of the probe and resonator. The probe was situated on a PLA component mimicking the robotic hand (including the metal backhand) and connected to the ANRITSU SHOCKLINE MS46524B Vector Network Analyzer (VNA). To assess the variation of the scattering parameters (S_11_) as a function of the probe and resonator alignment, a 3D-printed platform was created. This platform closely resembled the robot hand and a plastic object in terms of dimensions and material properties. All the 3D-printed components on the platform were made of PLA (ε_r_ = 3.55). The object, bearing the resonator, was linked to a stepper motor, which was controlled using an Arduino Uno R3 to automatically capture measurements for various angular alignments. In order to evaluate the performance of the sensor at various distances between the hand and the object in the *Y*-direction, both were placed on a linear rail. Three-dimensional-printed components were carefully designed to allow for adjustments in planar alignment along the *Z*- (up and down) and *X*- (left and right) directions. Additionally, two analog and digital displays were incorporated to precisely monitor and display the angle (around the *W*-axis) between the hand and the object.

During the experiment, the separation distance between the hand and the object was systematically adjusted, ranging from 8 mm to 60 mm with 4 mm increments. Additionally, measurements of the probe reflection coefficient (i.e., S_11_ scattering parameter) were collected by scanning over a 360-degree rotation with intervals of 18 degrees. These measurements were conducted under various scenarios, including situations where the resonator was positioned 10L, 10R, 10D, or 10U on the object, in addition to the central tag placement. In all experiments, the frequency range was configured to span from 0.5 GHz to 4.0 GHz. So as a result, data were collected for all angles at all distances and points.

### 3.2. Experimental Results

**Figure 7:**
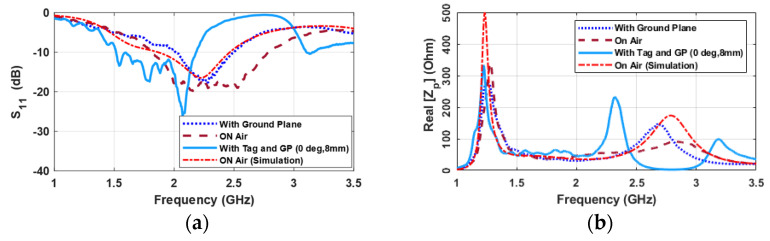
The exploration of the frequency characteristics of the probe and resonator for different conditions in terms of (**a**) reflection coefficient and (**b**) the real part of probe-input impedance.

The probe’s frequency response was analyzed by conducting an initial assessment in three distinct scenarios. These scenarios included the probe alone on air, the probe placed on the hand with a ground plane (metallic backhand), and on the hand with a ground plane along with the tag perfectly aligned at 8 mm. It is apparent in Figure 7a that the presence of the ground plane causes some minor alterations within the primary resonance bandwidth, which spanned from 2.0 GHz to 2.5 GHz, and a more pronounced change below 3.5 GHz (dotted blue vs. dashed red curves). Conversely, coupling with the tag (solid blue curve) resulted in massive modifications that are obvious both in S_11_ and impedance. Looking at S_11_, noticeable additional peaks between 1.5 GHz and 2.5 GHz and around 3.0 GHz have appeared. This frequency range warrants careful exploration to identify the most significant frequency for implementation in the studied grasping system.

The real part of the input impedance (Figure 7b) exhibits more pronounced variations under different conditions. When the probe is situated in front of the ground plane, there are slight fluctuations in the input impedance value around 1.25 GHz, along with an increase and frequency shift around 2.65 GHz. These observations underscore the influence of the ground plane on the probe impedance. When the probe is placed at 8 mm from the resonator, some interesting behaviors start to emerge. Initially, there is a slight rise in impedance accompanied by a frequency shift, indicating the impact of the resonator. However, at 2.34 GHz, a new peak point emerges, suggesting a strong connection with the resonator. Additionally, the peak points at around 3.1 GHz appear, which was around 2.65 GHz when the probe was positioned in front of the ground plane. These alterations can aid in the selection of suitable operating frequencies for different objectives and emphasize the significance of evaluating the system’s performance at various distances and angles. Overall, the data depicted in Figure 7 provide valuable insights into the impedance characteristics of the monopole probe under diverse conditions, facilitating informed decision-making for specific experiments or application goals.

## 4. Experimental Analysis for Automatic Alignment

A sensor response must be tested across different scenarios to identify the steps necessary to locate the object and determine the optimal grasping position. To achieve this goal, the data were categorized into four distinct sections. The first section involves motion spanning from 10 mm to the left to 10 mm to the right (i.e., along the *X*-direction). The second section encompasses movements from 10 mm downward to 10 mm upward (i.e., along the *Z*-direction). In the third section, data are analyzed for various distances along the *Y*-direction. Lastly, the alteration in the object’s angle relative to the hand is assessed for all positions. To make it easier to read and avoid repetition, we will use abbreviations for the positions: 10 mm to the left (10L), 10 mm to the right (10R), 10 mm downward (10D), and 10 mm upward (10U).

### 4.1. Correction and Alignment along X-Direction

**Figure 8:**
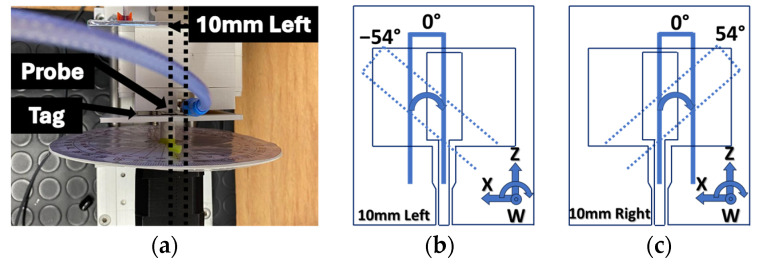
Alignment along *X*-direction: (**a**) setup configuration for orientation control, (**b**) resonator positioned 10 mm to the left, (**c**) resonator positioned 10 mm to the right.

In the initial scenario (Figure 8), the experiment entails positioning the hand in front of the object, deviating by 10L, 10R, and center. Furthermore, the angular orientation of the hand and the object is examined, spanning from −90 to 90 degrees (18-degree increments), with measurements being repeated at various distances (8 mm to 60 mm). In all these sets of measurements, the system was aligned in the *Z* direction. Moving the object to the left or right results in only one of the resonator arms being positioned in front of the central section of the probe. Additionally, in this configuration, symmetrical coupling does not occur during rotation. Also, Figure 8b,c illustrates how the resonator’s symmetrical design leads to a mirrored angular orientation when the hand is placed on either the left or right side. This means that placing the hand on the left side at an angle of -α degrees is equivalent to placing it on the right side at an angle of α degrees. It is important to note that during the tests, the hand rotation was always in a clockwise direction. Top of Form

**Figure 9:**
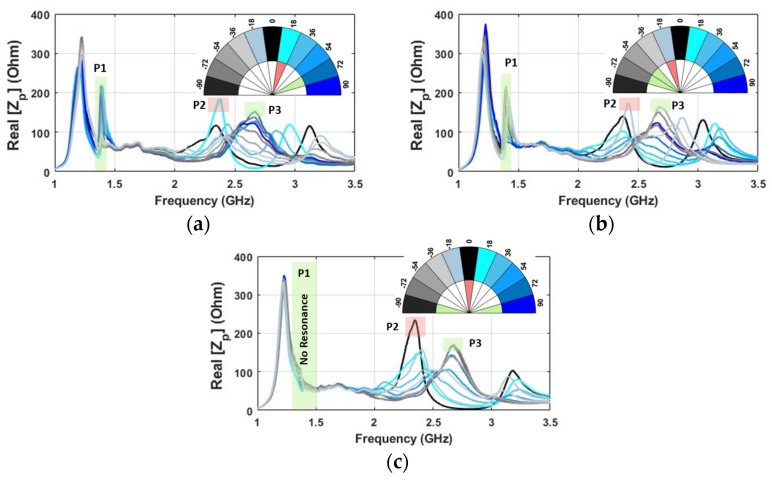
The real part of probe-input impedance (Z_p_) for various angles between probe and resonator in the *X*-direction when: (**a**) at 10 mm to the left (**b**) at 10 mm to the right and (**c**) at the center. Each color represents the corresponding angular misalignment.

The variations in the real part of input impedance at different angles, considering the placement of the resonator in three positions (center, left, and right, relative to the probe), are presented in Figure 9. It can be noticed that when the tagged object is positioned on the left or right side, an additional peak appears at a frequency of around 1.39 GHz due to the unsymmetric couplings, which are shown with P1. However, no significant peak is observed at this frequency when the resonator is aligned at the center. Moreover, the highest value of impedance belongs to angles of 54 and 72 degrees (after 0 degrees) when the resonator is placed on the left side. When the resonator is placed on the right side, the highest value of impedance is observed at approximately −54 and −72 degrees (before 0 degrees) because of the mirror effect. This behavior is clearly demonstrated at two distinct frequencies. The first frequency, labeled P1, is approximately 1.4 GHz, while the second frequency, labeled P2, is around 2.7 GHz. These frequencies have been highlighted in a vibrant green color to draw attention to them, allowing for easy identification and analysis. Another noteworthy finding is the presence of a resonance in the frequency range of 2.3 GHz–2.4 GHz (P2 and vibrant red), which is the main resonance that belongs to the resonator (Figure 7b) [23] and demonstrates distinct behavior depending on the position of the resonator. When the resonator is on the left, the maximum value of impedance is linked to an angle of 18 degrees (after 0), while it corresponds to −18 degrees (before 0) when the resonator is on the right side. Interestingly, this behavior further highlights that the peak at this frequency band is associated with 0 degrees when the resonator is at the center position. To facilitate comprehension of the observed behaviors, a 180-degree legend has been created for Figure 9. The legend is marked with colors that correspond to the frequency points, and the angles with the highest impedance at these frequencies are highlighted with the same color as the mentioned point. This design helps to visually identify the patterns of impedance at different frequencies and angles, providing a clearer understanding of the data. Furthermore, when the resonator is placed on either side, it induces an additional peak within the 2.8 GHz-to-3.2 GHz frequency range, while if it is positioned centrally, these peak points coalesce around comparable frequencies.

**Figure 10:**
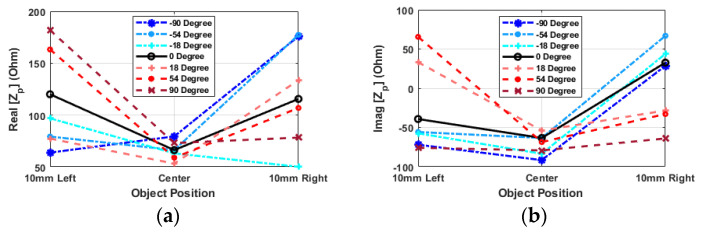
Input impedance of the probe (Z_p_) versus various alignments of probe and resonator in the X-direction (distance = 8 mm): (**a**) real part, (**b**) imaginary part. Frequency is equal to 1.39 GHz.

Figure 10 shows that the minimum impedance value is achieved at the center position, with a fixed frequency of 1.39 GHz in both the real and imaginary parts for most angles because there is no significant peak in this position. As the hand moves to the left or right, the impedance value increases. Moreover, the mirror relationship between positive and negative angles indicates similar impedance values for corresponding angles in the opposite side. Indeed, it can be observed that the impedance values for positive angles (indicated by red color tones) are higher when the hand is on the left side, while for negative angles (shown in blue), the impedance values are higher when the hand is on the right side. This asymmetry indicates that the position of the hand relative to the resonator has an impact on the impedance characteristics. At an angle of zero degrees, the real part of the impedance is undoubtedly almost identical on both the left and right sides, while it is significantly lower when the object is positioned at the center.

By analyzing Figure 9 and Figure 10 together, the peak point near 1.39 GHz indicates a misalignment of the hand. It has been observed that the alignment and positioning of the hand have a significant impact on the impedance behavior, particularly regarding the peak frequency presence at about 1.39 GHz. By shifting the hand slightly to the left or right, the center point can be identified as the imaginary part of impedance reaches the minimum value. Based on the increasing or decreasing behavior of the impedance value, it becomes possible to determine whether it is on the left or right side. When the hand is at zero degrees, the real and imaginary parts of the value reach their minimum at the central point.

**Figure 11:**
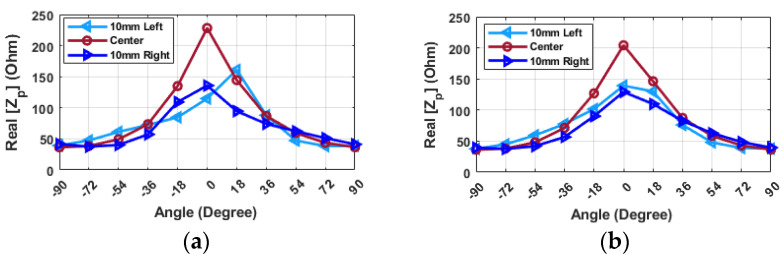
Input impedance of the probe (Z_p_) for various alignments of probe and resonator in the X-direction (f = 2.34 GHz): (**a**) distance = 8 mm (**b**) distance = 12 mm.

Figure 11 presents the probe-input impedance for various angular alignments of the probe and resonator in the *X*-direction when the frequency is fixed to 2.34 GHz. The actual impedance value undergoes a significant change when the hand is placed at the center. However, this value is lower and remains relatively constant when the hand is positioned either to the left or right. This pattern persists whether the hand is placed at 8 mm or 12 mm. Additionally, while the impedance value does not shift significantly with variations in distance when the hand is off-center, there is a substantial rise when moving from 8 mm to 12 mm for a zero-degree position.

**Figure 12:**
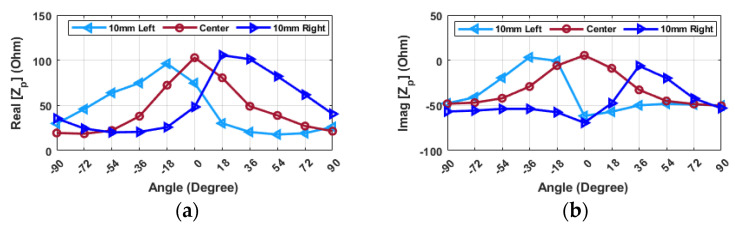
Input impedance of the probe (Z_p_) (**a**) real, (**b**) imaginary for various alignments of probe and resonator in the X-direction (distance = 8 mm, frequency = 3.18 GHz).

Furthermore, an alternative method for assessing the alignment from left to right is presented in Figure 12. In this figure, the input impedance at 3.18 GHz is depicted for various angles when the resonator is placed in different positions. It is illustrated that when the probe is positioned 10L, the impedance reaches its highest value at an angle of −18 degrees. Similarly, when the resonator is shifted 10R, the maximum impedance value is found at an angle of +18 degrees relative to the probe. This behavior is particularly noteworthy when the maximum impedance value is detected at angles of 0 degrees, indicating that the resonator is aligned at the center position. Although the changes for the center position are symmetric, the values of the real part for the left and right positions remain constant for angles greater than ±18 degrees. Moreover, the imaginary part of the impedance shows a similar pattern to the real part, but in the imaginary part, the maximum occurs at −36 and 36 degrees at left or right. In the imaginary part, the value is approximately −50 ohms for negative angles in 10R and for positive angles in 10L. After 0 degrees, the value increases until it reaches approximately ±36 degrees, and then decreases again for both 10L and 10R. However, for the center position, the change in values is symmetrical. It is worth noting that when the tag is not centered, the value at zero degrees is −50 ohms, while it is 0 ohms when the tag is at the center. Considering this characteristic, it becomes possible to accurately align the hand by first determining the angular alignment and then adjusting the position based on the impedance value. Understanding the relationship between impedance values and the alignment angle allows for effective hand alignment, particularly at 3.18 GHz. To locate the central position, the hand should be set to zero degrees, and a scan along the *X*-axis at specific frequencies should be performed, aiming to identify the maximum real part of the input impedance. This maximum value indicates the central position. To ascertain whether the hand is positioned on the left or right side, the hand should be adjusted within a range of plus or minus 18 degrees. Symmetrical changes in the impedance value signify that the hand is correctly placed at the center. By adhering to these guidelines, precise hand alignment can be achieved.

### 4.2. Correction and Alignment along Z-Direction

**Figure 13:**
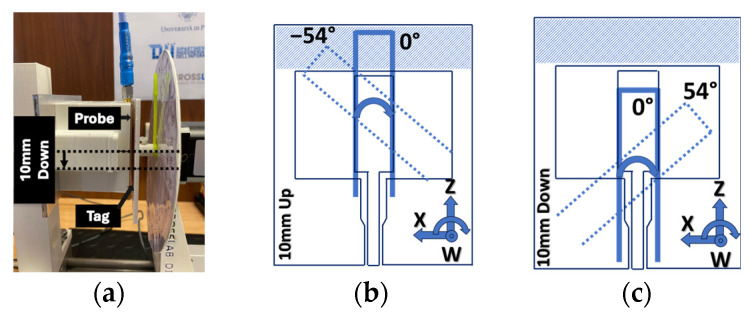
(**a**) Configuration for experimental *Z*-axis orientation control (**b**) schematic of resonator positioned 10 mm up (**c**) schematic of resonator positioned 10 mm down.

In a different scenario, the experiment aimed to determine the central position by vertically moving the hand along the *Z*-axis, as depicted in Figure 13. In this scenario, the performance of the sensor was assessed concerning angular variations and changes in distance. It is important to mention that when the resonator was positioned 10D, it was not directly in front of the upper side of the probe’s ground plane (hatched section in Figure 13c). This discrepancy could result in a decrease in the response of the resonator to the probe. However, when positioning it in 10U, the situation closely resembled the placement at the center (Figure 5c).

**Figure 14:**
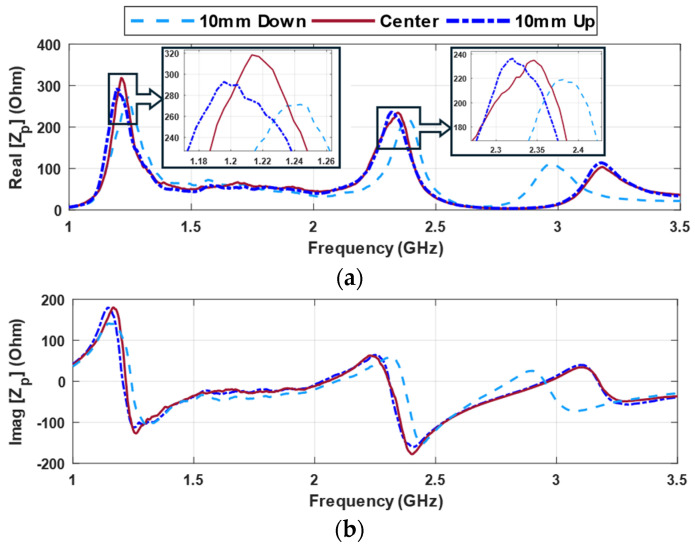
Input impedance of the probe (Z_p_) at frequency spectrum by different placement of the probe and resonator in the *Y*-direction at distance = 8 mm (**a**) real (**b**) imaginary.

When examining the input impedance values in Figure 14 with the resonator placed at the distance of 8 mm and zero degrees, it becomes apparent that the resonator’s position has a significant impact. Specifically, when the object is positioned either 10D, center, or 10U, noticeable shifts in impedance frequencies are observed. In a range of frequencies, such as around 1.2 GHz and 2.3 GHz, the impedance changes due to the 10 mm shift are quite discernible. Around the frequency of 1.2 GHz, the impedance value in the center position is higher compared to the misalignment case, and there is an approximately 15 MHz shift in frequency when transitioning the position. At frequencies near 2.3 GHz, the frequency shift is small as well but, due to the narrow curves, these small shifts are still helpful. However, a substantial discrepancy is observed around 3.0 GHz. Significantly, the resonant frequency measures 2.9 GHz when the label is 10D, while it increases to 3.18 GHz at 10U and when the object is at the center. Therefore, based on these observed frequency shifts, it is possible to determine, with a satisfactory level of accuracy, whether the resonator is located above the probe or not. This behavior provides a reliable means to identify the relative position of the resonator with respect to the probe. By selecting a specific frequency, it becomes simpler to assess the position of the object relative to the hand.

**Figure 15:**
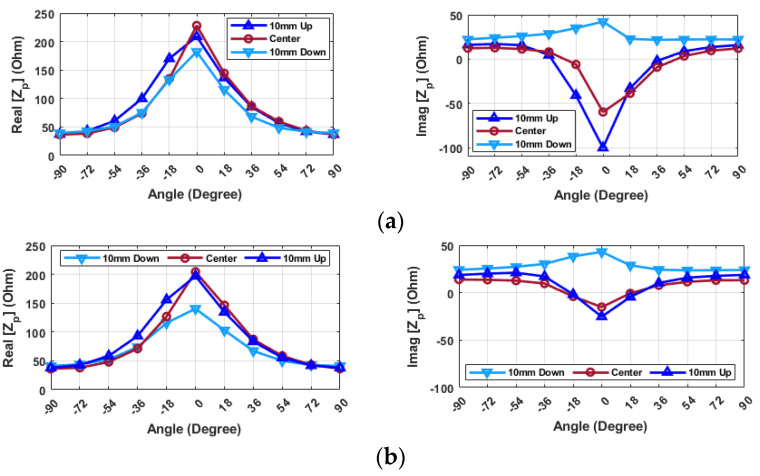
Input impedance of the probe (Z_p_) for various alignments of probe and resonator in the Y-direction (f = 2.34 GHz) (**a**) distance = 8 mm (**b**) distance = 12 mm.

The impedance values across various angles and three different positions in the *Z*-direction are depicted in Figure 15, with a focus on a frequency of 2.34 GHz. Initially, at this frequency, the real part of the impedance is noted to reach its highest value when the object is perfectly centered at zero degrees. The 10U position exhibits behavior similar to that of the center, though a marked decrease in value is observed for angles close to 0 degrees when the object is positioned 10D. Furthermore, the imaginary part of the impedance consistently stays positive when the object is placed below the hand, peaking at zero degrees and reaching its lowest point when the object is positioned 10U. The values at the center position resemble those at 10U, albeit with a slightly lower value for the 0-degree alignment. Shifting the distance from 12 mm to 8 mm results in similar behaviors, with some variations in values, especially at zero degrees.

**Figure 16:**
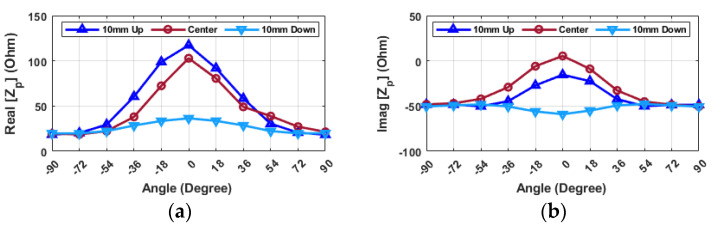
Probe (**a**) real (**b**) imaginary input impedance (Z_p_) for various alignments of probe and resonator in the Y-direction (distance = 8 mm, frequency = 3.18 GHz).

In another initial assessment, by selecting a frequency of 3.18 GHz (Figure 16), the real part of the impedance decreases as the probe moves either from 10U to 10D. Specifically, when the resonator is placed 10D, the impedance value exhibits minor fluctuations depending on the angle. Specifically, at angles of ±90 degrees, the real part and imaginary part of the impedance are approximately 20 ohms and −50 ohms, respectively. When the angle is zero degrees, with the hand and object aligned, these values shift to approximately 36 ohms for the real part and −59 ohms for the imaginary part. On the other hand, the value changes from 19 ohms to 117 ohms for the real part and from −48 ohms to −15 ohms for the imaginary part when the probe is positioned 10U. The value of the real part rises to approximately 100 ohms, and the imaginary part is 0 ohms in the case of alignment at the center. Therefore, to ascertain the alignment, it is feasible to search from the top to the bottom, and the maximum values in the imaginary parts of the input impedance can be used to determine the center point with a reasonable level of accuracy.

It is important to note that these value differences become noticeable when the angular positioning falls within the range of −36 to 36 degrees. Additionally, by the change of angle, the imaginary part of the impedance consistently remains below −50 ohms, and the trend decreases when the object is positioned 10D in the hand. When the hand is in the center position or 10U, the value of the imaginary part is higher than 50 ohms for all angles, and the trend increases. Also, when the hand is aligned at 0 degrees, the imaginary part of the impedance is at maximum and has a positive value if the hand is in the center position. However, in the case of the hand being placed in the 10D or 10U positions, the value stays negative across all angles.

### 4.3. Alignment along Y-Direction (Proximity Search)

**Figure 17:**
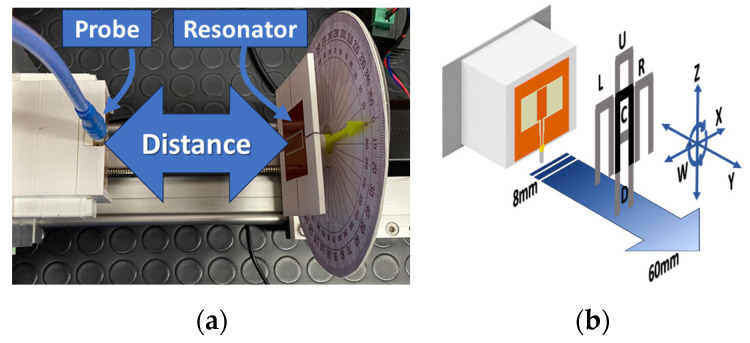
(**a**) Configuration for experimental *Y*-axis orientation control (**b**) schematic of resonator in different positions related to the probe.

**Figure 18:**
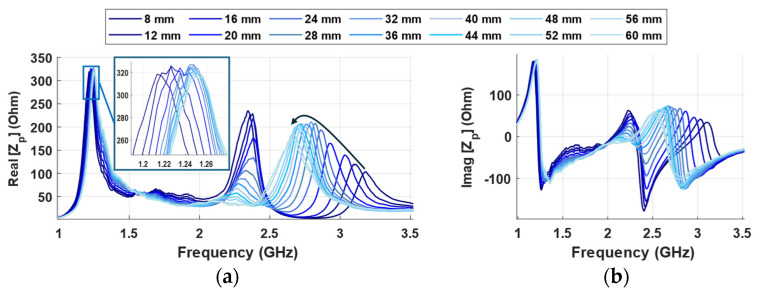
Input impedance of the probe (Z_p_), (**a**) real, (**b**) imaginary while adjusting the distance between the probe and resonator with angular alignment in 0 degrees.

To determine the distance and adjust the hand’s position appropriately for grasping an object (Figure 17), the system relies on monitoring changes in impedance at different distances. Figure 18 illustrates this process with the angle between the resonator and probe set at zero degrees, while the distance varies from 60 mm to 8 mm. At the initial resonance interval, small shifts in frequency are noticed as the distances change. This shift becomes more pronounced when the distance decreases to below 20 mm. For instance, when the distance is 8 mm, the resonance frequency is approximately 1.21 GHz. Increasing the distance to 20 mm results in a resonance frequency of around 1.24 GHz. Subsequently, as the distance is further extended up to 60 mm, the resonance peaks concentrate around 1.25 GHz, accompanied by a slight decreasing trend in impedance values.

In the second resonance interval, a markedly different behavior is evident. Firstly, the impedance value increases notably as the distance decreases. Additionally, a noticeable shift in the resonance frequency is found at 8 mm, which can serve as a confirmation of the optimal distance for grasping. Furthermore, the impedance value exhibits a slight increase up to 32 mm, followed by a more pronounced upward trend until 8 mm. This behavior aids the system in determining the appropriate distance by selecting a single frequency, which can be 2.34 GHz in this case, as a reference point. Moreover, there are additional peaks of impedance that span from 2.65 GHz to 3.18 GHz. Within this range, there is a noteworthy frequency shift, particularly for distances less than 32 mm. While the impedance value experiences a slight increase from 60 mm to 32 mm, it then undergoes a substantial decrease with significant variation until reaching 8 mm.

**Figure 19:**
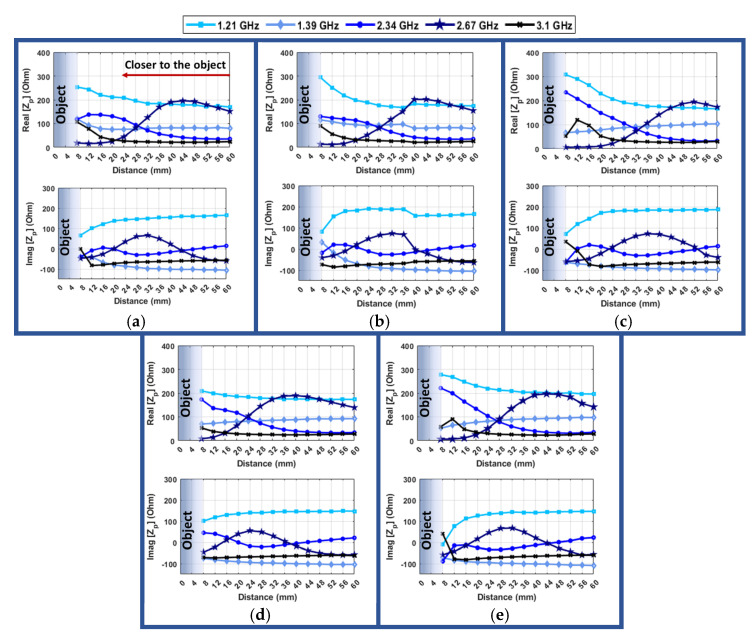
Real and imaginary parts of probe impedance (Z_p_) vs. distance between hand and object for different selected frequencies (for angle = 0 degrees) when the resonator is at (**a**) 10 mm left, (**b**) center, (**c**) 10 mm right, (**d**) 10 mm down, (**e**) 10 mm up.

To simplify the data and present it in a linear graph, the change in impedance at different distances is shown for four different frequencies, assuming an angular alignment of zero degrees between the resonator and probe (Figure 19). This figure allows for the selection of the best frequency for estimating the distance.

Overall, the trend of the changes in values for different frequencies is similar for different resonator-probe positions, which makes it possible to find the proper distance in all the situations. At a frequency of 1.21 GHz, both the real and imaginary parts exhibit a trend that can assist in determining the correct distance in all scenarios. In most cases, the real impedance values start around 200 ohms at 60 mm and increase up to approximately 300 ohms when reaching 8 mm. This variation is more pronounced for the case where the resonator is placed in 10D. On the other hand, the imaginary part demonstrates a decreasing trend as the hand gets closer to the object, and the variation in the value is smoother in most cases.

The change in impedance values, both in the real and imaginary parts, is not very noticeable in most cases when selecting a frequency of 1.39 GHz. The primary reason for this is that this peak appears only when the resonator is positioned to the left or right, and when this condition is met, there is an increasing trend in the impedance values from 16mm to 8 mm. However, in general, this change in the impedance value pattern may not provide significant assistance in determining the proper distance for grasping. 

When the frequency is set to 2.34 GHz, the real part of impedance shows a consistent and gradual increase with a high slope as the distance decreases for all cases. However, the imaginary part exhibits a different behavior, initially decreasing until it reaches 28 mm, after which it starts to increase until 16 mm, and then it once again starts to decrease until 8 mm in all cases, except when the object is positioned 10D. This change in the sign of the slope, along with the behavior of the real part of impedance, can be helpful in confirming distances of approximately 32 mm and 16 mm by passing through the local minimum and maximum points of the impedance curve. Indeed, it appears that the frequency of 2.34 GHz could be a promising choice for determining the proper distance for grasping due to its distinct impedance characteristics and the way it behaves across various distances and positions.

There are notable variations in the impedance value when considering a frequency of 2.67 GHz. Both the real and imaginary components initially increase and then exhibit a decreasing trend. The real part of the impedance rises until the hand reaches approximately 44 mm in all positions, after which it sharply declines, approaching zero when the hand is 8 mm away in every case. Meanwhile, the imaginary part starts from a negative value at a distance of 60 mm, increases to around 100 ohms at 28 mm, and then begins to decrease again, reaching a negative value once more at 8 mm in all positions. These significant variations, including changes in slope direction and the sign of the imaginary part, can aid the system in determining the optimal grasping distance.

At the frequency of 3.1 GHz, which is the peak frequency for the distance of 12 mm, the changes in impedance are very close together, and a significant increase is clear only after 20 mm in most cases. For the situation of center and 10U, there is a drop from 8 mm to 12 mm. This behavior is due to a shift in resonance frequency at different distances and can be avoided by selecting the resonance frequency of the desired distance, which is 8 mm in this case, of a resonance frequency close to 3.18 GHz. However, selecting the resonance frequency of 12 mm can be helpful by adding a confirmation point at 12 mm, which results in a significant decline in the impedance value after the hand passes this point, and it is noticeable by the change of the slope of the trend. The imaginary part in this frequency has a slight variation in most cases. When the object is in the center position, the imaginary value also shows an increasing trend after 16 mm. Indeed, it is possible to choose a frequency that corresponds to a distance less than 36 mm. Also, as each distance has a distinct peak frequency in this interval (2.5 to 3.5 GHz), adjusting the frequency to the desired value can ensure that the impedance value of the intended distance will be higher than all other distances. This approach allows for the precise control and measurement of distances between the hand and object.

It becomes then clear that frequencies of 2.34 GHz and 2.67 GHz are both useful for estimating distance and determining the appropriate distance for grasping. Additionally, when the hand is positioned on the left, right, down, or up, the behavior is very similar to the case when it is aligned at the center. This suggests that the system can still accurately determine the proper distance even if it is not perfectly aligned at the center.

**Figure 20:**
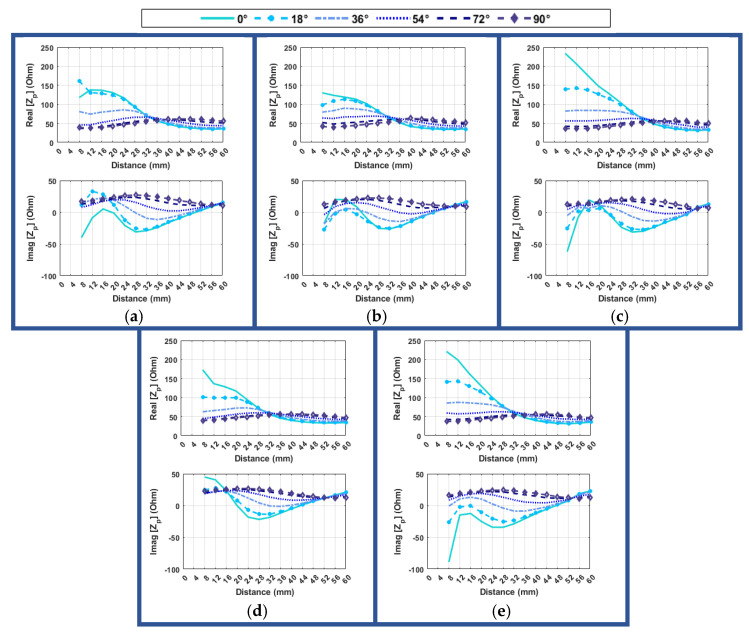
Variation of probe-input impedance (Z_p_) at 2.34 GHz with distance between hand and object for different angular alignments of hand and object, with the resonator at: (**a**) 10 mm left (**b**) center (**c**) 10 mm to the right (**d**) 10 mm down (**e**) 10 mm up.

It is also important to investigate the effect of changing the distance for different angles between the hand and the object. Figure 20 illustrates the variation in probe-input impedance at 2.34 GHz as the distance between the hand and the object changes, considering different angular alignments.

It is evident that more pronounced differences occur at various distances as the angle approaches zero degrees. Consequently, it becomes easier to distinguish between different distances when the angle is closer to zero degrees. Conversely, when the angle is between 90–72 degrees, the variation is negligible, thus making a precise estimate challenging. Therefore, if the impedance exhibits minimal variation, especially in its imaginary segment, it indicates that the angle of approach may not be optimal. However, for a value lower than 54 degrees, the distinctions become more discernible, and it generally becomes easier to accurately determine the proper distance. It appears that these conditions remain consistent regardless of the hand’s position in relation to the object. In general, for angles lower than 36 degrees, the real part of impedance typically exhibits an increasing trend, whereas the imaginary part initially decreases until 32 mm and then follows an increasing slope up to 16 mm, after which it begins to decrease again until 8 mm. However, for distances smaller than 10 mm, there is a distinctive pattern where the imaginary part decreases to 28 mm and then starts increasing again until 8 mm. These variations in impedance behaviors provide valuable insights for distance estimation and alignment considerations in different scenarios. The angle of alignment between the hand and the object significantly affects the system’s performance in estimating distance, and the patterns observed tend to be similar across different hand positions relative to the object. This highlights the significance of considering the angle of alignment when using the system for distance estimation.

**Figure 21:**
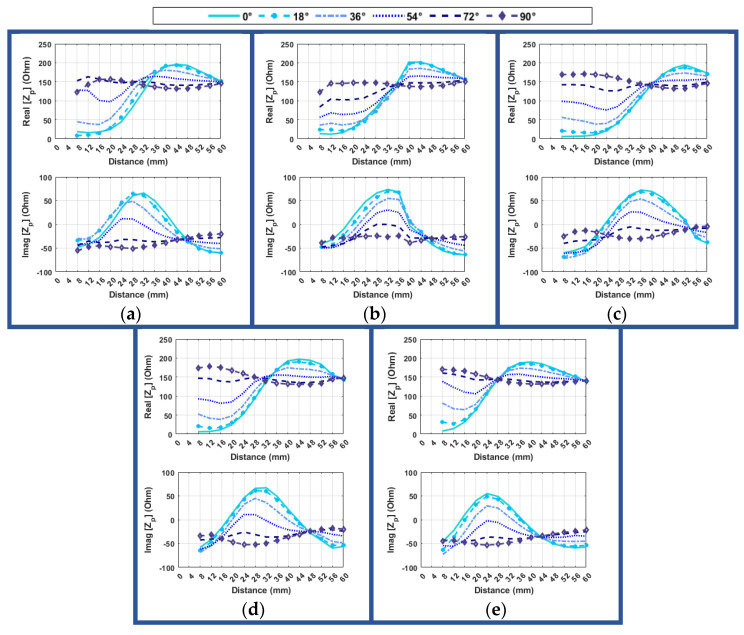
Variation of probe-input impedance (z_p_) at 2.67 GHz with distance between hand and object for different angular alignments, with the resonator aligned at (**a**) 10 mm to the left (**b**) center (**c**) 10 mm right (**d**) 10 mm down (**e**) 10 mm up.

The impedance values at the frequency of 2.67 GHz show bigger variations as a function of the distance, which makes it possible to estimate it for all scenarios if the angle is lower than 54 degrees between the hand and object (Figure 21). Even though the variation of the real part is higher compared to 2.34 GHz for angles of 90 or 72 degrees between the hand and the object, it still varies around 150 ohms for the real part and around −50 ohms for the imaginary part. In other words, a large angle and distance between the hand and the object indicate a non-ideal condition for grasping. This can be identified by specific impedance values (which are approximately 150 ohms for the real part and −50 ohms for the imaginary part in this case), with a low fluctuation around these values. When the angles drop below 54 degrees, both the real and imaginary parts of the input impedance show noticeable changes, increasing up to around 36mm and then dramatically declining. The decreasing slope of the real part becomes smoother for distances closer than 16 mm. These indicate the approach to ideal conditions. In this case, by observing an increasing trend in value, the impedance indicates that the hand is positioned farther than 36 mm from the object. Conversely, a decreasing trend in the value suggests that the hand is closer than 36 mm to the object. Additionally, a higher difference in the value of impedance as the hand gets closer to the object indicates a more proper angle for grasping. Furthermore, a smooth descending slope of the impedance value and a negative imaginary part indicate that the distance is less than 24 mm. In this case, an imaginary part value less than −50 ohms and a real part value close to zero suggest the optimal point for grasping the object.

Consequently, if the hand is not completely in front of the object, the information presented in Figure 18, Figure 19 and Figure 20 helps the system to accurately estimate the distance and determine the optimal grasping spot. The system can proficiently navigate toward the optimal distance while factoring in both the angle alignment and impedance behaviors. It should be noted that a sample object was tested, and the thresholds were determined for this object, which could potentially be generalized for a wider variety of objects.

### 4.4. Rotational Alignment (Angular Alignment)

The angular alignment process between the hand and object plays a crucial role in successfully grasping the object. In previous cases, it was observed that the process of finding the proper grasping position in all directions is easier when the hand is closer to zero degrees related to the object. The first step involves determining the most suitable frequency for this purpose, after which the focus shifts to achieving angular alignment between the probe and resonator.

**Figure 22:**
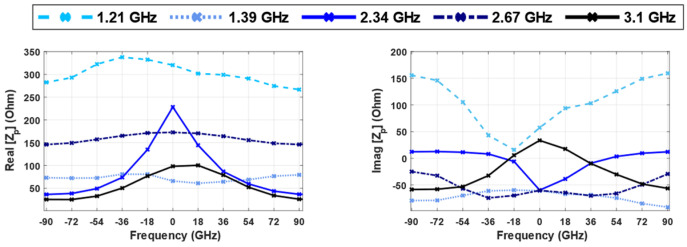
The probe-input impedance (Zp) vs. different angular orientations when the resonator is located at the center for different frequencies.

As it is presented in Figure 22, only two (i.e., 2.34 GHz and 3.1 GHz) of the five selectable frequencies (peak points in Figure 9) exhibit noticeable changes in real impedance values with varying angles. Moreover, at these two frequencies the probe imaginary input impedance drops to a negative value at zero degrees, which can help the system confirm the grasping point. The changes in the impedance value for the frequency of 3.1 GHz are more gradual to achieve an accurate angular alignment, and real and imaginary parts show a similar behavior. At 2.34 GHz, the impedance value exhibits the desired behavior with variations in the angle, where at angles of ±90 degrees, the value of impedance is at its minimum. As the hand rotates towards the alignment point, the impedance value increases, reaching its maximum value at 0 degrees, which is the optimal alignment for grasping the object. After selecting the desired frequency, it is necessary to observe how the sensor behaves at different distances and positions.

**Figure 23:**
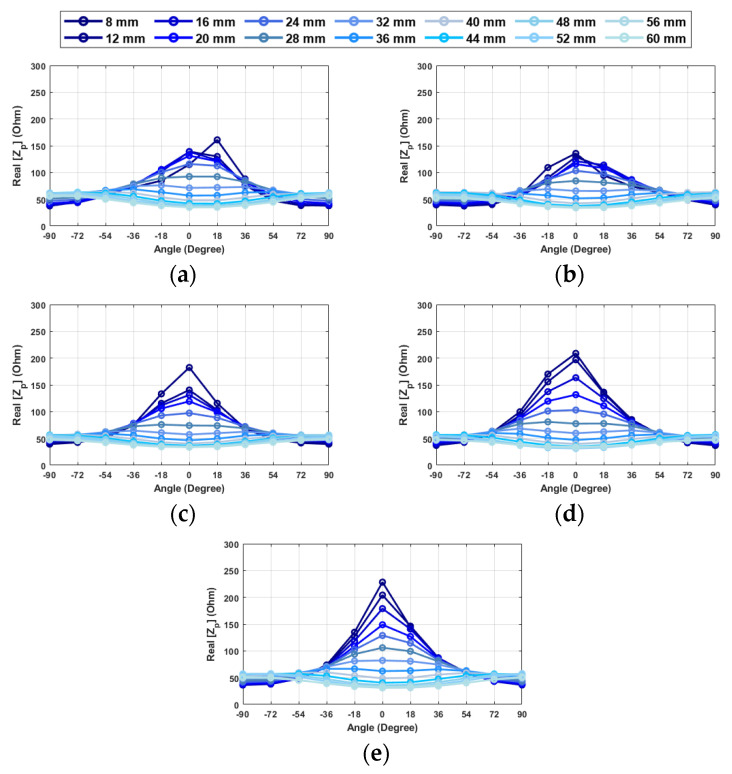
Real part of probe-input impedance (Z_p_) at 2.34 GHz vs. different angular orientations when the resonator is located at: (**a**) 10 mm to the left (**b**) 10 mm right (**c**) 10 mm down (**d**) 10 mm up (**e**) center (frequency: 2.34 GHz).

Figure 23 illustrates the actual part of the input impedance under various conditions. In many instances, when distances surpass 32 mm, the alterations in impedance values remain relatively consistent, allowing for alignment, albeit with potentially reduced precision. However, beyond this 32 mm threshold, there is a notable rise in the sensitivity of impedance changes, especially concerning distance. When the distance decreases, the differences between 0 degrees and adjacent angles become more pronounced. This condition is especially favorable when the distance is 8 mm, as it enables angular alignment with exceptional precision. It is also illustrated that angular alignment remains achievable even when the probe and object are not perfectly aligned along the *X*- or *Y*-direction. However, the values and differences between them are comparatively smaller in certain scenarios. For instance, when the object is positioned out of the center, the alignment angle can be estimated with an approximate value, and subsequent adjustments can be made after achieving full alignment.

By leveraging these observations, the system can navigate the angular alignment process with good accuracy, even in cases where the probe and object exhibit slight misalignment in the *X*- or *Z*-direction. This enhances the overall effectiveness of the system in accurately detecting and manipulating objects. Indeed, it is important to note that locating the zero angle at longer distances can be challenging or may result in reduced accuracy. Therefore, it is advisable to bring the distance closer and attempt the process again if the changes observed while rotating the hand are minimal. This approach ensures a more reliable and precise angular alignment, especially when combined with careful distance estimation.

## 5. Discussion and Considerations on the Alignment Procedure

**Figure 24:**
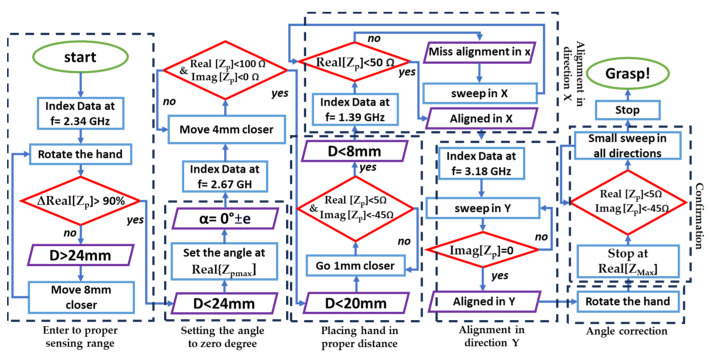
A comprehensive search and alignment strategy across four directions.

The presented results have allowed us to characterize the features of the proposed probe-tag configuration. The deployment of this sensor system requires a series of careful considerations aimed at defining a structured approach for successful object detection and manipulation in a scenario where the object’s location and orientation are unknown. A series of sequential operations, illustrated in the flowchart of Figure 24, is at the basis of the protocol to be followed for a reliable alignment and grasping operation. The exploitation of the individuated optimal frequencies as peculiar to the probe-sensor interaction is the key point for the overall search and discovery process. The only initial hypothesis for this proximity probe is simply to be within 60 mm from the tag. The search starts by moving the probe (i.e., the robotic hand) and collecting the data (i.e., the value of the probe-input impedance). Since having a good accuracy for the angle is challenging over long distances, the first goal is to bring the hand closer to the object and enter the range for accurate angular alignment (32 mm in this case). Based on the previously analyzed trends, the probe data collected at 2.34 GHz are the most useful for this task (Figure 23).

It is worth noting that a Vector Network Analyzer (VNA) provides the data within the addressed frequency range (i.e., 500 MHz–4 GHz), and other alignment procedures could be planned based on the previously analyzed trends. It is also important to note that the thresholds and values mentioned are specific to the test object in this study, and they may slightly vary due to the material and size of the object. The outcome of this first step is to bring the probe at a distance less than 24 mm from the object. At this distance, it is possible to achieve the alignment between the probe and the tag while still accepting a limited angular error. Next, the probe data collected at the other optimal frequencies (i.e., 2.67 GHz, 1.39 GHz, and 3.18 GHz) guide the hand closer and closer to the tag until a final correction is operated before the grasp takes place.

This iterative and systematic procedure based on the monitoring of the changes in the probe impedance allows for the performance of different operations, such as angle adjustment, distance search, and position corrections in the *Y-* and *X*-directions. This strategy can be adapted to other types of hands and tagged objects, provided that a proper initial characterization of the probe-input impedance behavior is addressed.

Finally, it is reasonable to assume that the time necessary to achieve the correct orientation with the tag ranges from 5 to 30 s, depending on the initial distance, angle, and position from which the system starts the operation.

## 6. Conclusions

This article introduces a novel wireless RF sensing system that uses a clipless passive tag to effectively grip plastic objects with robotic hands. The study showcases how the input impedance of a probe antenna can be utilized for aligning robotic hands. Specifically, the probe configurations have been designed to enhance the robustness of the sensing system and facilitate easy integration into robotic hands. To balance a strong response with compact size, a 3D-printed passive resonator in the shape of a hairpin has been implemented, allowing for seamless integration or direct printing onto objects. The evaluation of the sensing system was conducted using a specialized measurement setup. The study found that both the real and imaginary parts of the probe impedance provide sufficient data for aligning the hand in different directions. The measurement data indicate that this sensor system can determine the optimal angle and distance to a workpiece for successful grasping. In addition to determining distance, angle, and appropriate grasping points, the system can adjust its position in the planar direction for enhanced accuracy. The grasping strategy can be adapted through various steps as needed, demonstrating the system’s versatility in operating at different frequencies.

Finally, future work could enhance the sensing system’s capabilities by generalizing measurements for objects of different sizes and materials, both metallic and non-metallic. Adapting the system for metallic objects may need a complete and detailed investigation. Testing the system under more complex conditions, including varying planar and angular placements of the hand relative to the object, is also a promising area of research. Furthermore, incorporating machine-learning and numerical algorithms could improve the functionality of the sensing system. Ultimately, the system could be adapted to a robotic arm to perform real grasping operations on various objects.

## Data Availability

Data are contained within the article.
